# Real-World Clinical Outcomes of Treatment With Olaparib for BRCA1/2 Mutation-Positive Metastatic Breast Cancer in Japanese Patients

**DOI:** 10.7759/cureus.71522

**Published:** 2024-10-15

**Authors:** Kenichiro Tanaka, Junichiro Watanabe, Masami Arai, Tomoyuki Kushida, Tomoaki Ito, Koichi Sato, Mitsue Saito

**Affiliations:** 1 Department of Surgery, Juntendo University Shizuoka Hospital, Juntendo University School of Medicine, Izunokuni, JPN; 2 Department of Breast Oncology, Juntendo University Juntendo Hospital, Juntendo University School of Medicine, Tokyo, JPN; 3 Department of Clinical Genetics, Juntendo University Juntendo Hospital, Juntendo University School of Medicine, Tokyo, JPN; 4 Department of Breast and Endocrine Surgery, Juntendo University Juntendo Hospital, Juntendo University School of Medicine, Tokyo, JPN

**Keywords:** brca mutation, metastatic breast cancer, neutropenia, olaparib, real world

## Abstract

Background

Olaparib is effective in the treatment of metastatic breast cancer (MBC) patients, mainly confirmed by deleterious germline *BRCA *mutations. However, real-world data are scarce.

Methodology

A total of 10 cases from our institute and 17 cases from the relevant Japanese literature were reviewed. All 27 patients had MBC with confirmed deleterious germline *BRCA* mutations. Tumor reduction, response duration, disease-free interval (DFI), overall survival (OS), and safety were assessed. Additionally, exploratory research was conducted on the characteristics of a subgroup of patients who had a long-term response to olaparib.

Results

In the 10 cases from two Juntendo hospitals who received olaparib from July 2018 to December 2021, the median age was 48 years (range = 37-63 years). The same numbers of *BRCA1* and *BRCA2* mutations and the same ratios of luminal and triple negative (TN) breast cancer cases were observed. The metastatic sites, mainly visceral, included the lungs (n = 4), liver (n = 5), and bone (n = 6). The median duration of drug use was four months (range = 1-36 months). The median number of treatment regimens from metastasis to olaparib administration was 2.0 (range = 1-9 regimens). The median DFI was 21 months (range = 0-106 months). The median OS was not reached (range = 16-191 months). In the subgroup analysis of six cases in which olaparib was effective for four months or more, there was a case where DFI was longer than 100 months. Treatment-related toxicities (TRTs) included neutropenia (n = 5; 50%), anemia (n = 4; 40%), thrombocytopenia (n = 2; 20%), nausea (n = 1; 10%), and fatigue (n = 1; 10%). CTCAE (version 5.0) grade ≥3 TRTs included anemia (n = 1; 10%) and neutropenia (n = 5; 50%). On the other hand, neutropenia was previously said to be as low as 27.3%, lower than anemia (40.0%), in a population including various races. We additionally examined another 17 cases from the Japanese literature.

Conclusions

To our knowledge, this study reports the first real-world data of Japanese patients with MBC and confirmed deleterious germline *BRCA* mutations. Our data showed that olaparib is effective in the real world. The incidence of neutropenia seemed higher in Japanese patients.

## Introduction

Breast cancer (BC) is the most commonly diagnosed cancer in women worldwide and in Japan [1,2]. Hereditary breast and ovarian cancer is an autosomal dominant genetic disorder caused by germline pathological variants (PVs) in *BRCA1/2*. The prevalence of germline *BRCA1/2* deleterious mutations ranges from 1.2% to 8.8% in the unselected BC patient population [3,4]. In Japan, 1.45% of BC cases were reported to have *BRCA1* PVs, and 2.71% of BC cases were reported to have *BRCA2* PVs [5].

Olaparib (Lynparza®︎) is a molecular-targeted agent indicated for the treatment of “*BRCA* mutation-positive, *HER2*-negative inoperable or recurrent BC with prior cancer chemotherapy” and is an inhibitor of poly (ADP-ribose) polymerase, which repairs damaged DNA single strands, leading to the death of cancer cells. It is indicated for several malignancies, including BC, pancreatic cancer, ovarian cancer, and prostate cancer [6,7]. Although the efficacy of olaparib in the treatment of recurrent BC has been demonstrated in the OlympiAD trial and other studies [8,9], perhaps for the low frequency (1.2% to 8.8%) of cases with *BRCA1/2* PV among metastatic recurrent BC patients [1], there is no comprehensive real-world assessment in terms of its efficacy and safety. In the past few years, there has been an increasing number of case reports regarding olaparib use and its associated side effects. Thus, we considered it worthwhile to include sufficient data to be analyzed as real-world data of olaparib therapy.

## Materials and methods

In-house study (case series)

The medical records of metastatic breast cancer (MBC) patients with *BRCA* PV who received olaparib therapy in our institutes from July 2018 to December 2021 (“in-house cases”) were reviewed.

In-house cases were examined for age, *BRCA* pathological variants, BC subtype, stage, site of metastasis, number of regimens before the use of olaparib, the duration of response, disease-free interval (DFI), overall survival (OS), and safety (treatment-related toxicities (TRTs)). Median values were calculated and compared for each data point. The characteristics of a subgroup of patients who had a relatively long-term response to olaparib (i.e., duration of therapy (DT) ≥4 months) were also examined in an exploratory manner. We compared the population with the sub-group mentioned above.

Statistics

All statistical analyses were performed using JMP (version 17; SAS Institute, Cary, NC, USA). The results are expressed as the median (range). The age was also expressed as mean ± SD (range). The DFI was calculated from the date of resection of the primary tumor to the date of the first sign of tumor recurrence. The OS was calculated from the date of the diagnosis to the date of death from any cause. Data for patients who were still alive were censored at the date when the patient was last known to be alive. The Kaplan-Meier method was used to analyze OS in our cases.

Literature review

A literature search was conducted using the “Igaku Chuo Zasshi (ICHUSHI) web (https://www.jamas.or.jp/english/)” database, which is maintained by NPO Japan Medical Abstracts Society, to identify articles written in Japanese (“Japanese literature cases”). The search terms were as follows “Breast Cancer” AND “Olaparib” with articles restricted to “case reports and cases” and conference proceedings excluded. A total of 17 cases were extracted, and patient characteristics similar to the in-house study were examined. The characteristics of a subgroup of patients who had a relatively long-term response to olaparib (DT ≥4 months) were also examined in an exploratory manner, as the in-house study. We compared the populations and the subgroups of both the in-house study and the literature review.

The study was conducted in accordance with the latest version of the Ethical Guidelines for Medical and Biological Research Involving Human Subjects announced by the Japanese Ministry of Health, Labour, and Welfare (https://www.mhlw.go.jp/stf/english/index.html). Approval was obtained from the institutional review board of Juntendo University (approval number: E23-0128). Patients included in the study were allowed to opt out.

## Results

In-house study

We collected 10 cases of MBC with confirmed deleterious germline *BRCA* mutations in which olaparib was used at two Juntendo hospitals (located in Tokyo and Shizuoka, Japan) from July 2018 to December 2021. The mean ± SD (age range) and median age at the diagnosis of advanced disease were 48.3 ± 8.17 (37-63) and 49.0 years, respectively, and two of the 10 patients had de novo stage 4 disease. None of the patients had HER2 overexpression, and five patients were diagnosed with triple-negative (TN) breast cancer.

Five patients had *BRCA1* PV, while the others had *BRCA2* PV. Nine out of 10 patients showed visceral disease, and nine had multiple visceral metastases (Table 1).

**Table 1:** Patient characteristics of the in-house study. PV: pathological variant; TN: triple negative; PARP: poly (ADP-ribose) polymerase; DFI: disease-free interval; OS: overall survival

Case number	Age	Detected *BRCA* PV	Subtype	Stage	Site of metastasis	Number of regimens	Dosing period of PARP (months)	DFI (months)	OS (months)	Outcome
1	50	BRCA2	TN	II	Bone, liver	1	2	27	32	Deceased
2	48	BRCA2	Luminal	II	Bone, lung, liver, lymph node	9	9	106	191	Deceased
3	42	BRCA2	Luminal	IV	Bone, lung, liver	6	2	90	144	Alive
4	37	BRCA2	Luminal	II	Bone, liver, chest wall	2	1	48	75	Alive
5	37	BRCA1	TN	III	Lung, ovary, brain	2	4	2	31	Deceased
6	51	BRCA1	TN	IV	Lung, brain, meninges, lymph nodes	4	8	0	21	Deceased
7	48	BRCA1	Luminal	0	Ovary	1	36	35	36	Alive
8	50	BRCA1	TN	III	Pleura, pleural effusion, skin	2	3	0	16	Deceased
9	63	BRCA1	TN	II	Lung, lymph node	8	15	6	90	Alive
10	57	BRCA2	Luminal	II	Local, liver, bone, pleura, lymph node	5	4	15	52	Alive

As the majority of the patients who underwent olaparib therapy immediately after its approval had already received multiple lines of previous regimens, the median number of treatment regimens from metastasis to the administration of olaparib was 2.0 (range = 1-9 regimens), the median DT was 4.0 months (range = 1-36 months), and the median DFI was 21 months (range = 0-106 months).

**Figure 1:**
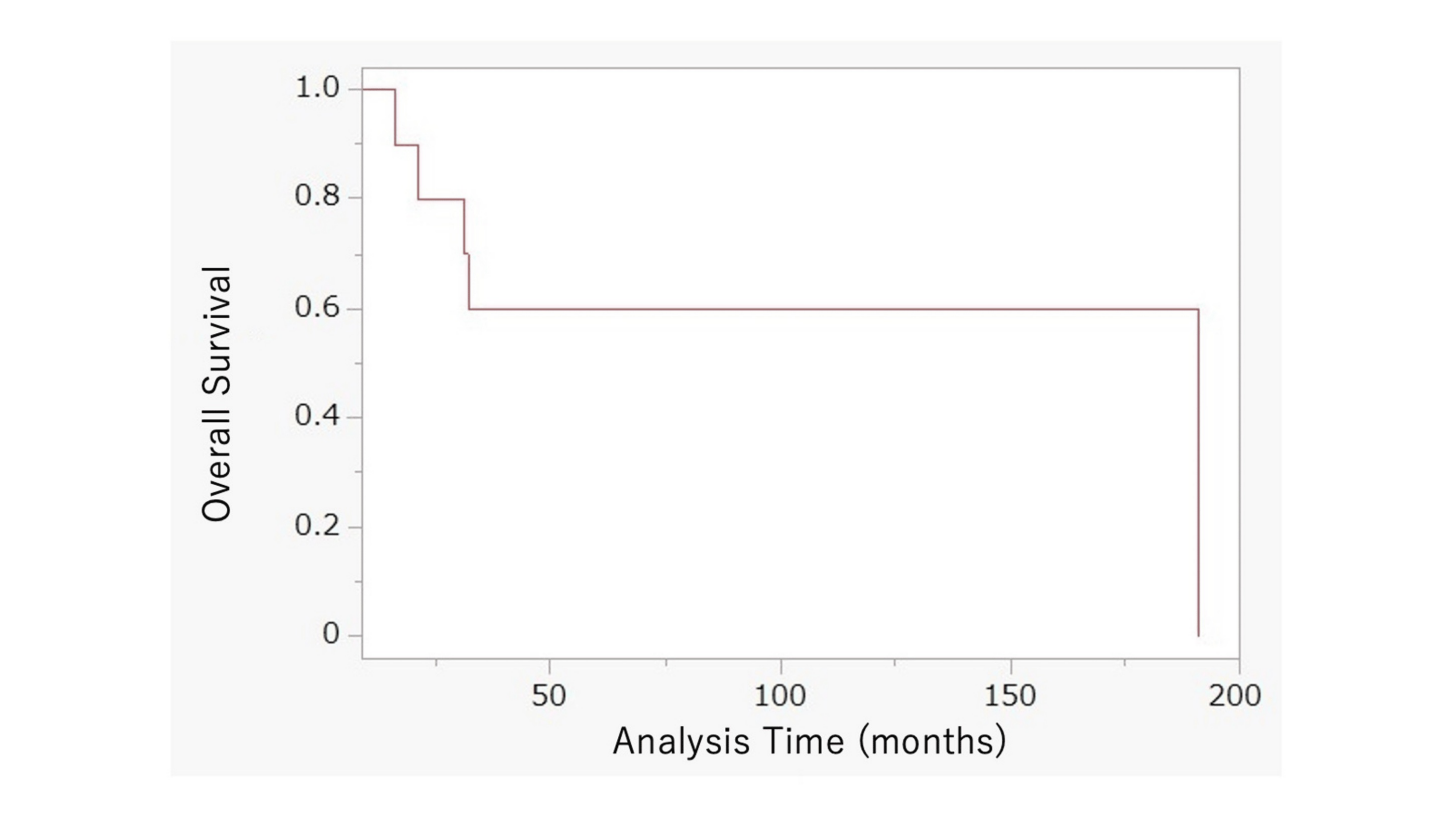
Kaplan-Meier curve for the overall survival of the in-house study The median survival time (50% survival rate) was not reached.

The median OS was not reached (range = 16-191 months) at the time of data cutoff according to the Kaplan-Meier curve. The two-year survival probability was 80.0% (Figure 1).

The characteristics of a subgroup of patients who had a relatively long-term response to olaparib (i.e., DT ≥4 months) were also examined in an exploratory manner. In this subgroup, there were six cases in which olaparib was effective for four months or more.

Four out of six patients had *BRCA1* PV (66.7%) and two had *BRCA2* PV (33.3%). The initial stages at the first diagnosis were relatively earlier than those in the overall population, and the breakdown of clinical stages at the time of the diagnosis of MBC was as follows: stage 0 (n = 1; 16.6%); stage I, 0 (0.0%); stage II, 3 (50.0%); stage III, 1 (16.6%); and stage IV, 1 (16.6%). Five out of six cases had two or more metastases at the time of the diagnosis of MBC. The median DFI of the subgroup was 10.5 months (range = 0-106 months). There was a case where DFI was longer than 100 months.

The TRTs that occurred in the population included neutropenia (n = 5; 50.0%), anemia (n = 4; 40.0%), and thrombocytopenia (n = 2; 20.0%) as hematologic toxicities, and nausea (n = 1; 10.0%) and fatigue (n = 1; 10.0%) as non-hematologic toxicities. The grade ≥3 TRTs (defined according to CTCAE version 5.0) included one (10.0%) case of anemia and five (50.0%) cases of neutropenia (Table 2). No other safety concerns were observed. In the subgroup where olaparib was effective for four months or more, the same tendency was observed. The rate of neutropenia was especially high in five out of six cases (83.3%).

**Table 2:** Treatment-related toxicities of the in-house study (n = 10). *: includes both hematological and non-hematological toxicities.

Treatment-related toxicities		
	Any grade	Grade ≧3
Any toxicity*	6 (60%)	5 (50%)
Anemia	4 (40%)	1 (10%)
Neutropenia	5 (50%)	5 (50%)
Thrombocytopenia	2 (20%)	0 (0%)
Fatigue	1 (10%)	0 (0%)
Nausea	1 (10%)	0 (0%)
Abdominal discomfort	1 (10%)	0 (0%)
Low blood pressure	1 (10%)	0 (0%)

Literature review

In total, 17 Japanese cases were identified from the database search. Data identical to those of in-house cases were extracted [10-24]. The median age of the patients was 50 years (range = 31-72 years). Eight (47.0%) patients had *BRCA1* PV, eight (47.0%) had *BRCA2* PV, and one (6.0%) had *BRCA* PV (gene name not described). Regarding the BC subtype, 10 (58.8%) patients showed the luminal type, and seven (41.2%) patients showed the TN type. Distant metastasis was more frequently observed (lung, n = 9; liver, n = 8; brain, n = 4; spinal cord, n = 1; bone, n = 3) than locoregional recurrence (lymph nodes, n = 11; chest wall/pleura, n = 4; skin/subcutaneous, n = 1; ipsilateral breast, n = 2; pericardium, n = 1; breast, n = 3). While 13 (76.4%) patients had multiple metastatic organs, one (5.9%) patient had bone-only metastasis.

The median DT was 5.0 months (range = 1.5-24.0 months), the median number of treatment regimens before olaparib administration was 2 (range = 1-7 regimens), and the median DFI was 36 months (range = 0-160 months) (Table 3).

**Table 3:** Patient characteristics of the literature review. PV: pathological variant; TN: triple negative; PARP: poly (ADP-ribose) polymerase; DFI: disease-free interval; OS: overall survival

Source	Age	Detected BRCA PV	Subtype	Stage	Site of metastasis	Number of regimens	Dosing period of PARP (months)	DFI (months)	OS (months)	Outcome
Osanai et al. 2019 [10]	71	BRCA2	Luminal	IV	Chest wall, lymph node	7	2	72	122	Alive
Yoneda et al. 2019 [11]	70	BRCA2	TN	II	Pericardium	1	12	37	42.5	Alive
Gomi et al. 2020 [12]	50	BRCA1	Luminal	III	Beast, chest wall, lung, pleura	2	4.5	120	236.5	Alive
Kin et al. 2020 [13]	44	BRCA2	Luminal	III	Lung	3	8	24	72	Alive
Kin et al. 2020 [13]	69	BRCA1	Luminal	II	Ovary, peritoneum, lymph node	2	4	160	192	Alive
Kohagura et al. 2020 [14]	51	BRCA1	TN	IV	Liver, bone	2	3	3	12	Deceased
Suzuki et al. 2020 [15]	34	BRCA1	TN	IV	Lung, brain, spinal cord	2	1.5	0	21.5	Alive
Yabe et al. 2020 [16]	43	BRCA1	TN	I	Brain, lung, lymph node, bone, liver	2	5	14	48	Alive
Arisawa et al. 2021 [17]	72	BRCA2	TN	III	Bone	2	12	24	42	Alive
Arisawa et al. 2021 [17]	35	BRCA2	TN	III	Lung, bone, lymph node	4	6	12	23	Alive
Nakagawa et al. 2021 [18]	67	BRCA1	Luminal	II	Breast, bone, lung, skin	6	10	120	370	Alive
Soyama et al. 2021 [19]	44	BRCA2	Luminal	II	Liver, lymph node	5	8	36	73	Deceased
Suzuki et al. 2021 [20]	31	BRCA1	TN	I	Lung, liver, bone, brain	1	3	35	38	Alive
Yamaguchi et al. 2021 [21]	50	BRCA2	Luminal	I	Lung, liver, bone, brain, lymph node	4	14	48	120	Alive
Yamamoto et al. 2021 [22]	49	unknown	Luminal	I	Liver	2	2	24	26	Alive
Abe et al. 2022 [23]	64	BRCA2	Luminal	I	Breast, liver, lung, bone, lymph node, chest wall	4	4	58	144	Deceased
Imai et al. 2022 [24]	36	BRCA1	Luminal	II	Breast, liver	1	24	36	60	Alive

Overall, 12 (70.6%) of the 17 cases in the literature review group showed a DT of ≥4 months of olaparib therapy. Among the olaparib responders, five (41.7%) had *BRCA1* PV, and seven (58.3%) had *BRCA2* PV. Regarding the BC subtype, eight (66.6%) cases were of the luminal type and four (33.3%) cases were of the TN type. The breakdown of the clinical stages at initial diagnosis was as follows: stage 1, 4 (33.3%); stage 2, 5 (41.7%); and stage 3, 3 (25.0%). The initial stages of the responders were less advanced in comparison to the responders of the in-house group. Nine (52.9%) of 17 patients had ≥2 involved organs. The median DFI of the subgroup was 36.5 months (range = 12-160 months). All three cases where DFI was longer than 100 months belonged to this subgroup.

We performed a subgroup analysis of patients for whom olaparib therapy was effective for more than four months, and six patients in the in-house study and 12 patients in the literature review were eligible for the analysis. Although the ratio of luminal and TN subtypes was equal to that in the in-house study, the luminal subtype tended to be dominant in the literature review. However, no differences were found in the *BRCA1/2* PV ratio or age between the subgroup and the in-house population.

The distribution of the metastatic sites of the subgroups was similar to that of the entire populations of both the in-house study and the literature review, with mostly visceral or lymph node metastases. Although the median DFI of the subgroup was not longer than the population, the cases where DFI was longer than 100 months (more than eight years) came from the subgroup of patients for whom olaparib therapy was effective for more than four months (DT ≥4 months), which was true both for the in-house study and the literature review.

The TRTs of the 17 cases in the literature review group included neutropenia (n = 5; 29.4%), anemia (n = 6; 35.3%) as hematologic toxicities, and nausea (n = 5; 29.4%), anorexia (n = 1; 5.9%), dysgeusia (n = 1; 5.9%), fatigue (n = 3; 17.6%), and interstitial lung disease (n = 1; 5.9%) as non-hematologic toxicities. Grade ≥3 TRTs (according to CTCAE version 5.0) included anemia (n = 2; 11.8%) and neutropenia (n = 3; 17.6%) (Table 4). No other safety concerns were found in the literature review. The same tendency, hematologic TRTs being dominant, was also observed in the subgroup. A comparison of the TRTs of the in-house study and the literature review found similar results.

**Table 4:** Treatment-related toxicities of the literature review (n = 17). *: includes both hematological and non-hematological toxicities.

Treatment-related toxicities		
	Any grade	Grade ≧3
Any toxicity*	10 (59%)	5 (29%)
Anemia	6 (35%)	2 (12%)
Neutropenia	5 (29%)	3 (18%)
Thrombocytopenia	0 (0%)	0 (0%)
Fatigue	3 (18%)	0 (0%)
Interstitial pneumonia	1 (6%)	0 (0%)
Nausea	5 (29%)	0 (0%)
Abdominal discomfort	0 (0%)	0 (0%)
Anorexia	1 (6%)	0 (0%)
Disgeusia	1 (6%)	0 (0%)
Low blood pressure	0 (0%)	0 (0%)

## Discussion

In the present study, we revealed that olaparib therapy in Japanese MBC patients with *BRCA1/2* PV showed sufficient clinical effectiveness. Regarding safety, concerning the TRTs, neutropenia for all grades was as high as 50% in the in-house study and 29% in the literature review of Japanese patients. Anemia was also frequent for all grades, especially grade >3, both in the in-house study and the literature review. Prior chemotherapy regimens, which may have contributed to neutropenia and/or anemia, were almost evenly distributed between 0~7 regimens for the cases of the in-house study, and 1~2 for all cases of the literature review.

In the OlympiAD study, a head-to-head comparison of olaparib and treatment of physician’s choice (TPC) was conducted. The study included 302 patients with unresectable/metastatic BC with *BRCA1/2* PV. The median progression-free survival (PFS) was significantly longer in the olaparib group than in the TPC group (7.0 months vs. 4.2 months; hazard ratio (HR) = 0.58; 95% confidence interval (CI) 0.43-0.80; p < 0.001) [8]. The median OS was numerically longer in the olaparib arm than in the TPC arm; however, the difference was not statistically significant (19.3 vs. 17.1 months; HR = 0.90; 95% CI = 0.66-1.23; p = 0.513) [9,25]. In the LUCY study, a real-world setting phase IIIb study of olaparib versus TPC, the median PFS in the olaparib arm was 8.18 months; however, 54% of the patients received olaparib as first-line therapy for metastatic disease [26].

While our in-house data showed almost identical patient backgrounds, such as the proportion of *BRCA1/2* PV and subtype, to those two studies, the median DT of 4.0 months was shorter than that of the two studies. Regarding the two-year survival rate, the OlympiAD and phase IIIb studies showed survival rates of 36.6% and 51.1%, respectively, while that of our in-house data was 80.0%. However, the number of cases in our study was small; thus, a direct comparison with these two studies is difficult [9,26]. Thus, from the viewpoint of PFS, olaparib should be administered at an earlier stage of metastatic disease.

The two-year survival rate of 80% in the in-house study could be due to the median DFI of 21 months, which was almost two years. It is well known that recurrent BC patients with a longer DFI have a better prognosis than those with a shorter DFI [27].

Comparing TRTs in our study with the OlympiAD study and LUCY study, there was a tendency for more frequent neutropenia, especially among those with grade ≥3 TRTs, in both the in-house study and the literature review, in comparison to the two studies. Japanese patients may be more vulnerable to neutropenia when using olaparib than non-Japanese patients. Despite data on chemotherapy, there are several reports that Asians, including Japanese patients receiving chemotherapy, are more vulnerable to neutropenia than other races [28,29]. The frequency of anemia was almost the same between the OlympiAD study, the LUCY study, and our study; about 40% of the patients had anemia and >10% of the patients had grade ≥3 anemia.

In the OlympiAD study, although the patient population was early BC, not MBC, the TRTs of the subset of Japanese patients differed from the global population. Both neutropenia (32.8% vs. 16%) and anemia (45.3% vs. 23.5%) were more frequent in Japanese patients compared to the global population. Both grade ≥3 neutropenia and anemia were also more frequent in Japanese patients (more than 10% of the Japanese patients) than in the global population (less than 10% of the global population) [30].

Concerning the background of the patients who had neutropenia, all of the patients, both the in-house study and the literature review, were in the subgroup in which the DT of olaparib was >4 months. Stages were distributed between 0~Ⅳ, and there were both luminal type and TN cases. Longer DT of olaparib may have contributed to the higher frequency of neutropenia.

Our study shows the possibility that patients with a longer DFI may have a longer DT of olaparib, which would lead to a better prognosis. We can also say from our study that Japanese patients who undergo olaparib treatment should be carefully monitored for TRTs, especially neutropenia. Appropriate dose reduction and dosing interval adjustments are required.

This study had some limitations, including its retrospective nature and relatively small sample size. However, the clinicopathological features of the study corresponded fairly well with those of the OlympiAD study.

## Conclusions

To our knowledge, this is the first real-world report of Japanese MBC patients with confirmed deleterious germline *BRCA* mutations treated with olaparib. The therapeutic effect, in terms of improving OS, was almost identical to that reported in previous global studies. However, neutropenia was more frequently observed. Further studies with more patients are needed to reveal the details of the real-world utility, efficacy, and safety of olaparib in Japanese MBC patients with *BRCA1/2* PV.
